# Diastolic shock index (DSI) works… and it could be a quite useful tool

**DOI:** 10.1186/s13613-020-00728-x

**Published:** 2020-08-06

**Authors:** Gustavo A. Ospina-Tascón, Glenn Hernández, Jan Bakker

**Affiliations:** 1 Department of Intensive Care Medicine, Fundación Valle del Lili–Universidad Icesi. Cali, Cali, Colombia; 2 Traslational Medicine in Critical Care Medicine and Experimental Surgery Laboratory (TransLab-CCM), Universidad Icesi, Cali, Colombia; 3 Departamento de Medicina Intensiva, Pontificia Universidad Católica de Chile, Santiago, Chile; 4 Department of Intensive Care Adults, Erasmus MC University Medical Center, Rotterdam, The Netherlands; 5 Department of Pulmonary and Critical Care, New York University, New York, USA; 6 Division of Pulmonary, Allergy, and Critical Care Medicine, Columbia University Medical Center, New York, USA

Dear Editor

We thank Dr. Dalmau for his interest in our recent manuscript [1]. Vasodilatory shock is fundamentally characterized by a failure of peripheral vascular smooth muscle cells to constrict, resulting in altered tissue perfusion and organ dysfunction. Although severe inflammation is perhaps the most prominent factor triggering vasodilatory shock, other non-inflammatory-related mechanisms could also be implied. Arterial hypotension is the natural consequence of decreasing arterial tone and represents one of the cardinal signs of vasodilatory shock along with a progressively impaired response by the vascular smooth muscle to endogenous circulating and exogenous vasoconstrictors. Nevertheless, hypotension observed during septic shock results from a complex interaction between relative and absolute hypovolemia, myocardial dysfunction, vasodilation, and altered blood flow distribution. In addition, alteration in the balance among sympathetic, cholinergic and anticholinergic inflows can directly affect inflammatory and immunologic response beyond their direct effects on the heart and vessel walls.

Clinicians facing septic shock clearly recognize low diastolic arterial pressure (DAP) as a classical sign of vasodilation, which is thought to be explained, at least in part, by lowering vascular tone. In normal conditions, vascular tone of the resistance arteries and arterioles determines peripheral vascular resistance, contributing in turn to the regulation of blood pressure and blood flow to, and within the tissues. Vascular tone, defined as the activation degree of vascular smooth muscle cells, provides a measure of vascular regulation in response to external stimuli as hormonal, myogenic, neural and endothelial-derived factors. Nevertheless, direct in vivo assessment of vascular tone during dynamic clinical conditions is not quite evident and it has been typically based on indirect measurements of vascular resistance: blood flow; arterial pressure/volume, pressure/diameter, and compliance/distensibility–pressure relationships [2, 3].

In general, an index is the ratio of one dimension of a thing (such as a physiological parameter) to another dimension. As such, diastolic shock index (DSI) represent a simple ratio between DAP and heart rate (HR), as a magnitude possibly reflecting how severe cardiovascular dysfunction is. DSI is based on three simple sequential thoughts: (a) under isovolemic conditions and constant arterial compliance, shortening of diastolic time is associated with higher DAP while a prolonged diastole leads to an opposite effect; (b) acute reductions in arterial pressure are normally compensated by increased sympathetic activity, which usually leads to tachycardia; (c) consequently, simultaneous and opposite variations in DAP and HR could reflect more severe cardiovascular alteration, with progressively high HR unable to compensate DAP drops as consequence of a gradual increased vasodilation. Interestingly, even though Dr. Dalmau suggests that DSI should not represent vascular tone because [times^−1^].[mmHg] units do not correspond to any mechanical property of the vascular walls (a situation with which we fully agree), he supports the presence of a direct link, or dependence, between DAP and arterial vasomotor tone.

Operational definitions of shock have classically included the reduction of mean (MAP) and/or systolic arterial pressure (SAP), assuming the pivotal role of both MAP and SAP on organ perfusion, in addition to the clinical prognostic value of sustained low MAP values. Nevertheless, although DAP is not widely mentioned and it is not considered to categorize the severity of shock, evaluation of DAP could have significant clinical implications especially when the underlying mechanism of shock is vasodilation. Although admittedly, hypotension observed during septic shock results from a complex interaction among altered pump, “pipes”, and volume factors, as previously discussed.

Peripheral resistance, arterial compliance, pulse wave velocity, and the timing of pulse wave reflections affect both steady (i.e., the mean arterial pressure) and dynamic components (i.e., the systolic and diastolic pressures) of arterial pressure. In turn, arterial compliance is affected by the blood pressure changes per se, and vascular biomechanics dependent on the composition of vessel walls. Admittedly, alterations in vascular tone should also influence SAP as correctly suggested by Dr. Dalmau. Nevertheless, changes of arterial pressure after fluid loading in septic “fluid-responders” usually translates into SAP increases with minimal or no effect on DAP. Certainly, one classical characteristic of resuscitated vasodilatory shock is the increased pulse pressure, which obeys to the stroke volume rise with no immediate variations in peripheral resistance. Indeed, classical observations demonstrated that variations of pulse pressure are greater when compliance changes at constant resistance than when resistance changes at constant compliance. Importantly, when cardiac output increases, the arterial compliance determines the rate at which the mean arterial pressure will attain its new, elevated value but will not determine the magnitude of the new pressure.

The arterial system has a dual function: first, as a simple conduit to adequately supply blood to the tissues; and second, as a converter of pulsatile flow generated by the heart beating into a continuous flow of blood at the periphery (i.e., the Windkessel phenomenon). In his letter, Dr Dalmau correctly signals, according to the two-element Windkessel model, that as the DAP is mainly determined by the diastolic time constant RC (i.e., arterial resistance times compliance), one could interpret it as being representative of the arterial tone, while the dependence of systolic arterial pressure on the arterial RC is more directly confounded by other parameters—namely, heart function parameters—than the DAP is. Nevertheless, the Windkessel model does not determine diastolic pressure itself (although it clearly influences it).

Recent experimental and observational data suggest that a very early start of vasopressor support could be beneficial [4, 5]. Nevertheless, there are no clear signals indicating when vasopressor support should be started. In this way, very early signals of severe vasodilation should alert on its possible immediate requirement and as such, DSI could be a quite useful tool on this purpose.

## Data Availability

Not applicable.

## References

[1] Ospina-Tascón GA, Teboul JL, Hernandez G, Alvarez I, Sánchez-Ortiz AI, Calderón-Tapia LE (2020). Diastolic shock index and clinical outcomes in patients with septic shock. Ann Intensive Care.

[2] Guerrisi M, Vannucci I, Toschi N (2009). Differential response of peripheral arterial compliance-related indices to a vasoconstrictive stimulus. Physiol Meas.

[3] Zheng D, Allen J, Murray A (2007). Non-invasive in vivo assessment of changes in peripheral arterial properties with estimation of arterial volume compliance. Physiol Meas.

[4] Byrne L, Obonyo NG, Diab SD, Dunster KR, Passmore MR, Boon AC (2018). Unintended consequences; fluid resuscitation worsens shock in an ovine model of endotoxemia. Am J Respir Crit Care Med.

[5] Ospina-Tascón GA, Hernandez G, Alvarez I, Calderón-Tapia LE, Manzano-Nunez R, Sánchez-Ortiz AI (2020). Effects of very early start of norepinephrine in patients with septic shock: a propensity score-based analysis. Crit Care.

